# GlasVent—The Rapidly Deployable Emergency Ventilator

**DOI:** 10.1002/gch2.202000046

**Published:** 2020-09-06

**Authors:** Adamos Christou, Markellos Ntagios, Andrew Hart, Ravinder Dahiya

**Affiliations:** ^1^ Bendable Electronics and Sensing Technologies (BEST) Group University of Glasgow Glasgow G12 8QQ UK; ^2^ Glasgow Royal Infirmary NHS Greater Glasgow and Clyde Glasgow G12 0XH UK; ^3^ CMVLS The University of Glasgow Glasgow G128QQ UK

**Keywords:** 3D Printing, covid‐19, DIY, healthcare technology, ventilators

## Abstract

As a result of the novel Coronavirus disease (COVID‐19) outbreak, a surge is witnessed in the demand for mechanical ventilators needed for treating affected patients. With the rapidly virus spreading around the globe, the shortage of ventilators becomes a global challenge and numerus efforts are followed. While industry mobilizes toward producing medical grade equipment, a number of low‐cost and less complex emergency ventilators have been developed, mainly through academic and open‐source channels, with a hope to meet any temporary needs gap until medical grade ventilator provision becomes sufficient. Herein, the design and implementation of one such emergency ventilator called GlasVent is presented, which an automated version of manual resuscitator device, commonly known as big valve mask or artificial manual breathing unit bag and widely used prior to initiating the mechanical ventilation. GlasVent uses 3D printed mechanical parts, widely available materials and off‐the‐shelf electronic and sensing devices which can be fast assembled. Furthermore, it requires minimal training and can be operated manually by hands or legs, thus meeting the emergency requirements even in the low‐resource settings or regions with less developed healthcare systems. Post‐COVID‐19, such ventilators can potentially find use in clinical care of a wider variety of patients with injury, pulmonary noncommunicable diseases, and severe asthma etc.

## Graphical abstract

The increase in the demand for ventilators due to rapid spread of novel coronavirus and the resulting pandemic is a global challenge. This paper presents an emergency ventilator called GlasVent, which uses 3D printed parts, widely available materials and off‐the‐shelf electronics and sensing devices. The GlasVent ventilator can be operated automatically or manually by hands or legs.

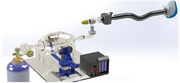

## 1 Introduction

The novel Coronavirus disease (COVID‐19) pandemic's rapid expansion has led to the exponential increase in the numbers of cases and deaths worldwide. When SARS‐CoV‐2, the virus that causes COVID‐19, gets in the human body, it comes into contact with the mucous membranes that line our nose, mouth, and eyes, and infects the upper or lower part of respiratory tract. As a result, the respiratory tract and lungs swell, become irritated and inflamed and, in some cases, the infection can reach all the way down into alveoli, where oxygen goes into the blood and carbon dioxide comes out. For those who develop trouble breathing, medical care outside of the home is needed.

**Figure 1:**
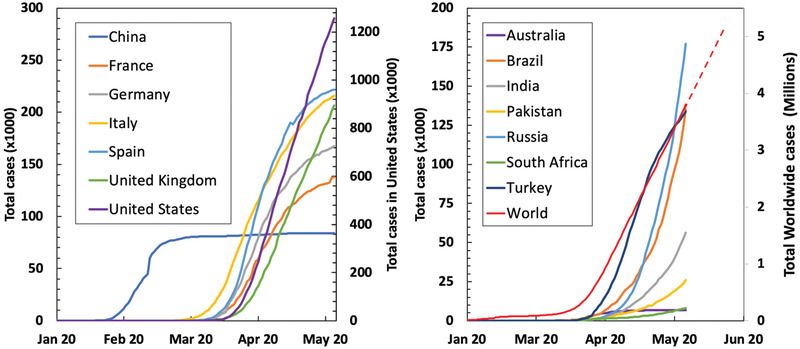
Number of reported cases of Covid‐19 in countries across the globe. The worldwide trend suggests a steady increase in cases in the near future. Source: Our World in Data.

Breathing is fundamental to life and it is regulated by a complex system of checks and balances in the body. The biological equilibrium shifts into a state of respiratory failure when the respiratory mechanism is compromised by infection due to virus or by other respiratory diseases. When this occurs, the mechanical ventilator (MV) becomes an essential life support, which must also protect the lungs from further damage. It provides positive airway pressure and airflow to support work of breathing, sustain oxygenation, and enable patient recovery. That is why MVs are nowadays coveted machines. While numbers of COVID‐19 patients are rapidly rising, the shortage of ventilators is of dire concern and may be compounded by shortage of professionals who can manage ventilators, while increasing numbers of healthcare workers are becoming infected, hospitalized, or succumbing to the disease. Although most countries are affected, the negative impact on low‐middle income countries (LMICs) is likely to be higher, as they often lack sufficient equipment and their critical care systems are in their infancy. By studying the numbers of reported cases in various countries around the globe^[^ ^1^ ^]^ (**Figure** **1**), it is apparent that countries which were first hit by the pandemic are currently in a remission stage with the number of daily new cases stabilized or falling. However, a large number of countries, many of them in the developing world, are at earlier stages of the epidemic. Less capable healthcare systems, fewer conducted tests, less effective isolation measures, and second waves could result in an exponential increase in cases in the coming months and subsequently an increased demand for ventilation equipment.

The increased demand for ventilators has resulted in various efforts and initiatives to mitigate the supply shortage.^[^ ^2^ ^]^ Established manufacturers of ventilators are reallocating resources to increase production of the essential equipment while also making some of their designs freely available so that others can try to reproduce them.^[^ ^3^ ^]^ In some other cases, companies with significant experience and skilled personnel that operate in other engineering fields are trying to replicate and adapt existing designs of ventilation equipment to be able to manufacture them at their facilities.^[^ ^4^, ^5^ ^]^ While the above endeavors aim to produce ventilators that are closer to the current standard medical grade equipment, another wave of initiatives is focusing on producing low cost emergency ventilators suitable for fast deployment. The key characteristics of these devices are the use of generic off‐the‐shelf parts, widely available materials and simple fabrication techniques. These devices offer only some of the capabilities of conventional ventilators and would be justifiable in emergency cases were the availability and cost of standard medical equipment is limiting. Here, we discuss some of these emergency devices along with detailed design and implementation of one such device, i.e., GlasVent, which an automated version of manual resuscitator device, commonly known as big valve mask (BVM) or artificial manual breathing unit (AMBU) bag and widely used by clinicians prior to initiating the mechanical ventilation. GlaVent requires minimal training for operation and unlike other emergency ventilators it can also be operated manually (e.g., by hands or legs), if needed.

## 2 Mechanical Ventilation: Key Parameters

**Figure 2:**
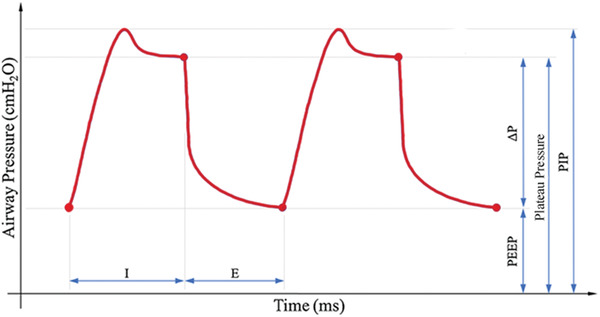
An idealized ventilator breath cycle highlighting key pressure parameter including positive end expiratory pressure (PEEP), driving pressure, peak inspiratory pressure (PIP), and plateau pressure. The two‐time components, i.e., inspiration I) and expiration E) are also shown.

Mechanical ventilators are medically focused machines and could be complexed to operate for anyone with nonclinical background. For better comprehension by nonclinical readers, and to offer entry points from the engineering perspective, it is important to understand the fundamental parameters at play. Thus, here we cover the key parameters in a simplified mechanical ventilator that should be regularly monitored. These include tidal volume, airway pressure, peak inspiratory pressure, plateau pressure, positive end‐expiratory pressure, and respiratory rate.^[^ ^6^ ^]^ The tidal volume is the air volume entering and exiting the lungs each breath. Excessive volumes can overinflate and stretch lung tissue causing injury. It is therefore chosen by the clinician, usually using predicted body weight. The airway pressure is the pressure supplied from the ventilator to the patient during ventilation. There are four distinct measures of airway pressure during a typical mechanical ventilation breathing cycle (**Figure** **2**): a) Positive end expiratory pressure (PEEP)—an important setting used to maintain lung recruitment needed for gas‐exchange and to prevent the collapse of terminal lung units (atelectasis), which can cause further damage; b) Peak inspiratory pressure (PIP)—the maximum airway pressure during inspiration; c) Plateau pressure, which is usually lower than PIP and measured during an end of inspiratory pause, represents the pressure in the alveoli and is often used as a threshold for high‐pressure levels; and d) Driving pressure, which is the pressure difference added above PEEP to plateau pressure. These four measures of airway pressure provide the extremes of both inspiration and expiration (Figure 2) and together with the time components, this set of metrics can roughly describe the entire breathing cycle with only minimal redundancy. Besides various pressure values, the respiratory rate is another important parameter which reflects the number of breaths per minute during ventilation. It is commonly around 16–20 so that each breath is ≈3–4 s in length. Setting the respiratory rate together with tidal volume ensure adequate minute ventilation (liters of air per minute).^[^ ^7^, ^8^ ^]^

**Figure 3:**
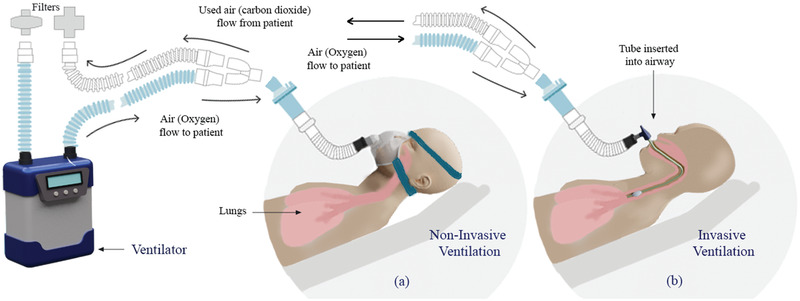
The general scheme of a) Noninvasive, and b) Invasive methods of ventilation.

The delivery method of mechanical ventilation could also vary depending on the condition of patient. The two possible modes (**Figure** **3**a,b) for delivering mechanical ventilation are noninvasive and invasive, determined by the intubation of the patient or not.^[^ ^9^ ^]^ The ventilation modes can be further subdivided into control or support modes, depending on the patient's breathing efforts and sedation, and finally, pressure or volume‐controlled modes. Noninvasive ventilation is delivered via face mask, whereas, invasive ventilation involves insertion of a laryngeal mask, endotracheal tube, or tracheostomy, as shown in Figure 3a,b. Invasive ventilation is typical when the ventilator is required to manage the entire patient work of breathing. The application of noninvasive ventilation for acute respiratory distress syndrome is not an ideal solution, but in an emergency situation, such as the one related to COVID‐19, this mode can provide useful complementary support. Furthermore, noninvasive ventilation is actively being improved.

The first step prior to placing a patient on any mode of ventilator is the bag mask ventilation (commonly known as BVM or AMBU bag). The AMBU bag is a widely available medical device which mainly consists of a soft air reservoir which is manually pressed to supply air to the patient. The patient receives positive pressure ventilation through squeezing of the bag by a healthcare professional. After squeezing, the bag is able to return to its original shape due its purposefully selected material. In this case, the oxygen is administered via tubing from this bag. This is a form of external ventilation, as the patient is being ventilated by an outside force. The bag mask ventilation can theoretically support a person's airway indefinitely, but it is used as a temporising measure (e.g., during patient transport or induction of anaesthesia) until a safer, more definitive airway is secured. With the recent surge in ventilator demand resulting in limited availability, such devices could be used for extended periods of time to provide external ventilation in emergency situations. The use of automated version of these devices that provide limited functionality compared to conventional mechanical ventilators due to lack of better alternative is what we term here as the emergency ventilation.

## 3 Rapidly Deployable Ventilators Developed in Response to COVID‐19

Among various designs of emergency ventilators that emerged during the past few months, many revolve around the automatic operation of the manual bags or resuscitator device. The manual resuscitator is commonly used in scenarios where patient ventilation is needed urgently, and mechanical ventilators are not available. The devices are primarily meant to be used for short time (up to few hours) as they understandably lack meaningful control over some of the important ventilation parameters discussed in Section 2. However, various designs of automated manual bags now being explored aim to introduce some control over these parameters by automatically actuating the BVM so that they could be used for longer periods. The control is open loop in its basic form, while the use of sensors allows for closed loop operation and enables a kind of assistive ventilation through patient feedback. Some of these devices are discussed below and compared in **Table** **1**.

**Table 1:** Key characteristics of mechanical and emergency ventilators

Ventilator type	Key characteristics
Mechanical Ventilators	• Monitoring and precise control over a large number of parameters associated with patient ventilation status • Robust and reliable operation over long periods with minimal supervision requirements • Ability to detect changes in patient's condition and warn the medical staff or perform automatic adjustments to ventilation mode • High cost and significant time required for manufacturing due to the use of complex and purposefully made components • Extensive training is required to operate the system
Emergency Ventilators	• Monitoring of limited breathing parameters necessary to perform basic ventilation support • Reliability and robustness in operation does not match mechanical ventilators • Limited capabilities in detecting and warning for changes in patient's condition thus requiring further monitoring by medical staff • Low cost and minimum manufacturing time by using off‐the shelf components to achieve rapid deployability even in low resource settings • Simple operation requiring minimal training

**Figure 4:**
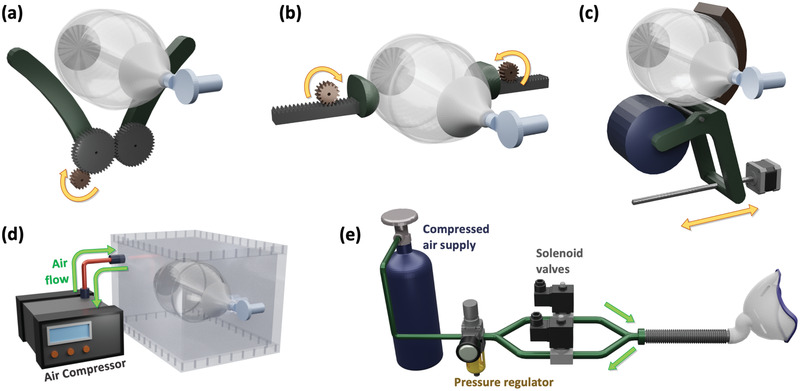
Various mechanisms of emergency ventilation systems developed by: a) MIT, b) RICE University, c) OpenLung, d) Oxford University and King's College London, and e) Sample configuration of a pneumatic emergency ventilator system.

One example of emergency ventilators is E‐Vent, which employs a motor driven gear mechanism that operates a pincer‐like structure to squeeze the AMBU bag as a human hand would (**Figure** **4**a).^[^ ^10^ ^]^ This design uses off‐the‐shelf aluminium parts and machined gears along with laser cut acrylic. Different versions of this designs are being explored to adapt to alternative manufacturing methods. The system also includes a pressure sensor to monitor airway pressure. As of writing this article, the E‐Vent team at Massachusetts Institute of Technology (MIT) is testing their prototype and are providing design information and results on their website, where the information on ventilation and working of the manual resuscitator are also provided.

Another example an automated BVM‐based emergency ventilator, developed by RICE University, uses a dual motor rack and pinion mechanism to operate an AMBU bag (Figure 4b).^[^ ^11^ ^]^ With mainly laser cut and 3D printable parts, this system is do‐it‐yourself (DIY) friendly. The design also includes an on‐board liquid crystal display (LCD) and controls, while pressure sensing is to be incorporated in future versions. Yet another initiative called Open Source Ventilator with a collaboration of teams in Ireland and Canada has developed a system based on a leadscrew mechanism to actuate the AMBU bag from one side.^[^ ^12^ ^]^ This system includes pressure sensing as well as flow rate sensing at the inlet and outlet, while there is also provision for backup power supply. All three of the aforementioned approaches use a motorized mechanism to actuate the BVM, where the speed of the motor controls the breathing rate. The motors operate in reciprocal motion and the motion range is proportional to the tidal volume. The control for reciprocal motion of motors requires complex electronics and could increase the cost of device.

An alternative approach for automating a manual resuscitator has been demonstrated by Oxford University and King's College London, UK.^[^ ^13^ ^]^ The system (Figure 4d), called OxVent, uses compressed air to actuate the AMBU bag, which is placed inside a sealed plastic enclosure. The compressed air is pumped in and removed from the enclosure in a controlled way via solenoid valves. This approach eliminates the need for moving parts. The unique way of compressing the AMBU bag could provide some pressure feedback without the need for an extra sensor. However, the design relies on electro‐pneumatic components and this does seem to reflect on the increased estimated cost.

There are also reports of emergency ventilators which are not based on the manual resuscitators. For example, a team at Imperial Collage London has developed a system that only uses pneumatic components (solenoid valves and pressure sensors) to achieve ventilation.^[^ ^14^ ^]^ A similar approach was recently highlighted by Ennomotive as a potential solution for a rapidly scalable ventilator system.^[^ ^15^ ^]^ A set of solenoid valves and pressure gauges are placed inside a table‐top enclosure with on‐board controls. These pneumatic‐based systems more closely resemble the operation of conventional mechanical ventilators and have the potential to be chained together for use in multiple‐patient wards. These approaches require the use of off‐the‐shelf pneumatic components which do not perform at the level of medical‐grade equipment and are subject to regional availability. Another factor to consider is the requirement for compressed air supply which should be available at hospitals but not granted in other cases. **Table** **2** summarizes the key characteristics of the mechanical and emergency ventilator types.

**Table 2:** Comparison of emergency ventilator systems

	Mechanism	Materials	Parameter control	Other features /highlights	Manual	Battery	Cost estimate
E‐Vent (MIT, USA)	Automated BVM with Gear system; Motor with reciprocal motion; home positioning needed; Bag: dual side operation	Laser cut acrylic; Waterjet aluminum plates; CNC; Gears can be difficult to source or manufacture	Tidal volume; Breathing rate; Pressure sensing; Volume and rate controlled by range and speed of motor	Sturdy construction; Extensive details for plumbing; BVM easily swappable	n/a	n/a	n/a
ApolloBVM (Rice, USA)	Automated BVM with Rack pinion mechanism; Dual motors (servo) which require synchronization; Bag: dual side operation	Laser cut acrylic and wood; 3D printed parts; Metallic gears, which can be difficult to source; 3D printed plastic may be unreliable	Tidal volume; Breathing rate; Pressure sensing (pending); Volume and rate controlled by range and speed of motor	On‐board controls and LCD; DIY friendly; Off‐the shelf‐parts; BVM easily swappable	n/a	n/a	< $300
Open Source Ventilator/ OpenLung (Ireland, Canada)	Automated BVM with leadscrew mechanism; Motor with reciprocal motion; Bag: Single side operation (increases wear)	Laser cut acrylic; Some specialty parts (not much info given); Leadscrew: complex arrangement, difficult to source and fabricate	Tidal volume, Breathing rate, Pressure sensing; Flow sensing (in and out); Volume and rate controlled by range and speed of motor	On‐board controls and LCD; BVM easily swappable (Not clear if prototype exists yet)	n/a	Yes	$100
OxVent (Oxford, KCL UK)	Automated BVM with Compressed air Electropneumatic system; Requires compressed air supply/compressor, solenoid valve	Perspex; Pneumatic fittings; Perspex box needs to be well sealed; Subject to availability of pneumatic components	Tidal volume; Breathing rate; (possible pressure sensing‐indirect); Volume and rate controlled by supply and release of compressed air	No moving parts; On‐board controls and LCD; BVM not easily swappable	n/a	n/a	£1000
Emergency Ventilator (Imperial College, UK)	Pneumatic system; Electropneumatic system; Requires only solenoid valves and pressure sensors	No need for custom parts; Subject to availability of pneumatic components	Tidal volume; Breathing rate: Intrinsic pressure control; Air supply controlled directly with valves	Not complete (next version follows a similar approach)	n/a	n/a	£1500
Ennomotive (Frede Jensen) Spain	Pneumatic system; Electro‐pneumatic system; Requires only solenoid valves and pressure sensors	No need for custom parts; Subject to availability of pneumatic components	Tidal volume; Breathing rate, Intrinsic pressure control; Air supply controlled directly with valves	Detailed and complex design, suitable for multiple patient arrangements	n/a	n/a	£350
Our Design‐ GlasVent (Univ. Glasgow, UK)	Automated BVM with slider crank mechanism; Motor (Stepper): Continuous one directional rotation; Bag: dual side operation	Laser cut acrylic; 3D printed parts; Metallic rods; No custom metallic parts; no gears; parts easily available in supply chain	Tidal volume; Breathing rate: Pressure sensing; Rate controlled by speed of motor; Volume controlled manually	Off‐the‐shelf parts; DIY friendly; BVM easily swappable; Acrylic case letting patient to view moving parts	Yes	Yes	<£200

## 4 GlasVent

**Figure 5:**
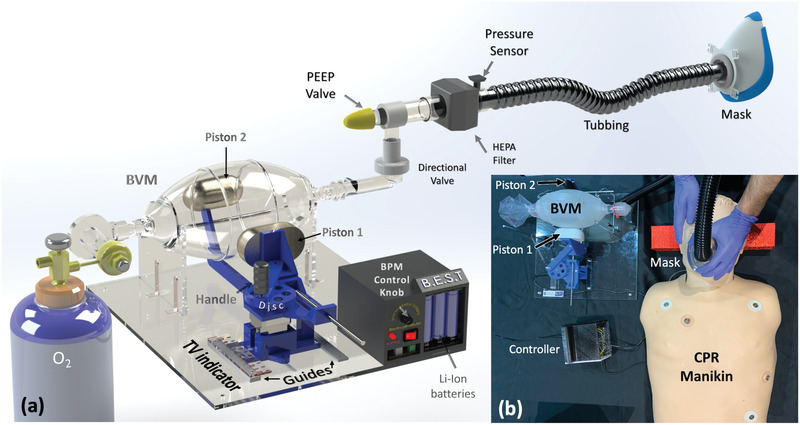
GlasVent System. a) CAD model of GlasVent assembly. b) System testing on a medical mannequin.

**Figure 6:**
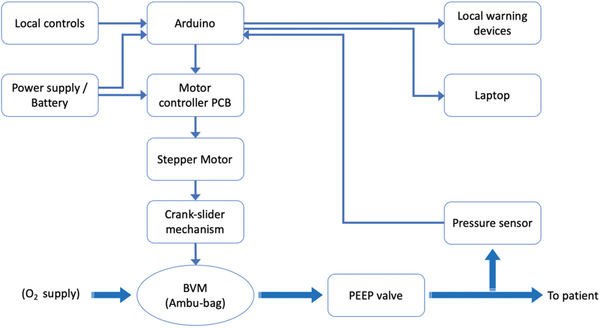
GlasVent Block diagram.

**Figure 7:**
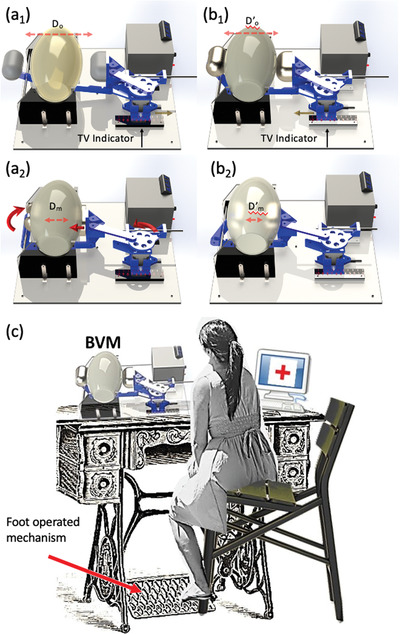
GlasVent adjustable maximum Tidal Volume. a) The base slid away from the BVM resulting in smaller compression. b) The base was moved toward the BVM resulting in the increase of Tidal Volume. c) Scheme for potential foot operated variant.

The GlaVent, developed by University of Glasgow team, is an affordable and rapidly deployable emergency DIY ventilator (**Figure** **5**) which takes into consideration most of the basic requirements for ventilators. The system utilizes a crank—slider mechanism to convert the rotation motion of the motor to reciprocal linear motion.^[^ ^16^ ^]^ The base of the motor is able to slide toward or away from the BVM to regulate the maximum compression of the bag and provides control over the tidal volume exerted by the system. As discussed in Section 2, the tidal volume is an important parameter to be controlled as it can vary between patients depending on their age, and physical condition, etc. The GlasVent system has been designed keeping in view the specifications for emergency ventilators outlined by regulatory authorities, such as department of health and social care, as may be noted from the block diagram (**Figure** **6**), which shows most of the critical components. The main driving force of the design was fast deployment, easily scalable, simple to assemble, low‐cost, and manual operation in emergency. Additionally, the system is designed with ease of use in mind as the end users should be able to operate the machine with minimum training. The system has three operating schemes: a) mains or supply operated; b) battery operation, and c) manual operations via a small handle attached to the rotating disc of the system. This latter feature is distinct as it enables the system to operate when mains power or batteries are not available. The system could also be adapted for operation by foot instead (e.g., fitting with sewing machine, see **Figure** **7**c). This mode is possible because the system is designed to operate with continuous one‐directional rotation rather than reciprocal motion found in other emergency ventilator designs. A detailed description of various mechanical and electrical/electronic components is given below.

### 4.1 Mechanical

GlasVent utilizes a crank—slider mechanism to convert the rotation motion of the motor to reciprocal linear motion (Piston 1). An extended lever arm allows for a second piston (Piston 2) to also move reciprocally along a curved path but in opposite phase. As a result, the two pistons are moving toward each other and the BVM located in between is compressed (Figure 5). The drive train begins with a disc attached centrally to a stepper motor. A link structure is attached to the disc at distance of 23 mm from the center of the disc. The link is 125 mm long and connects the Piston 1 support structure. The support structure is connected to a rod that is partially constrained so that it can only move in a linear direction toward and away from the bag. These three basic components constitute the crank‐slider mechanism and are able to convert rotation motion to linear. The support part is connected to another large link underneath the BVM which is in turn connected to piston 2 lever. The piston 2 lever is allowed to pivot on a rod at its center. These four parts (Piston 2 lever, large link, rod, Piston 1 support structure) transfer the linear motion of the Piston 1 support structure to a reciprocal partial rotation motion that lead to compression of BVM from the opposite side of Piston 1. This is required as a single piston is not able to sufficiently squeeze the BVM and supply the required volume of air to the lungs. Also, the symmetrical deformation of the BVM from both sides reduces the wear on the bag and increases its lifespan. This prototype also uses a small spring attached to a rod placed vertically to the base. The other end of the spring is attached to the connection between the disc and the link (not shown in Figure 5). The spring and the link form a 90° angle when the pistons fully compress the BVM. While the motor is retracting the piston, the spring extends and stores the energy. At the time of compression of the BVM the spring contracts and releases the stored energy to support the motor. The base of the motor is able to slide toward or away from the BVM (Figure 7a,b). This regulates the maximum compression of the bag and provides control over the tidal volume exerted by the system.

### 4.2 Electronics

**Figure 8:**
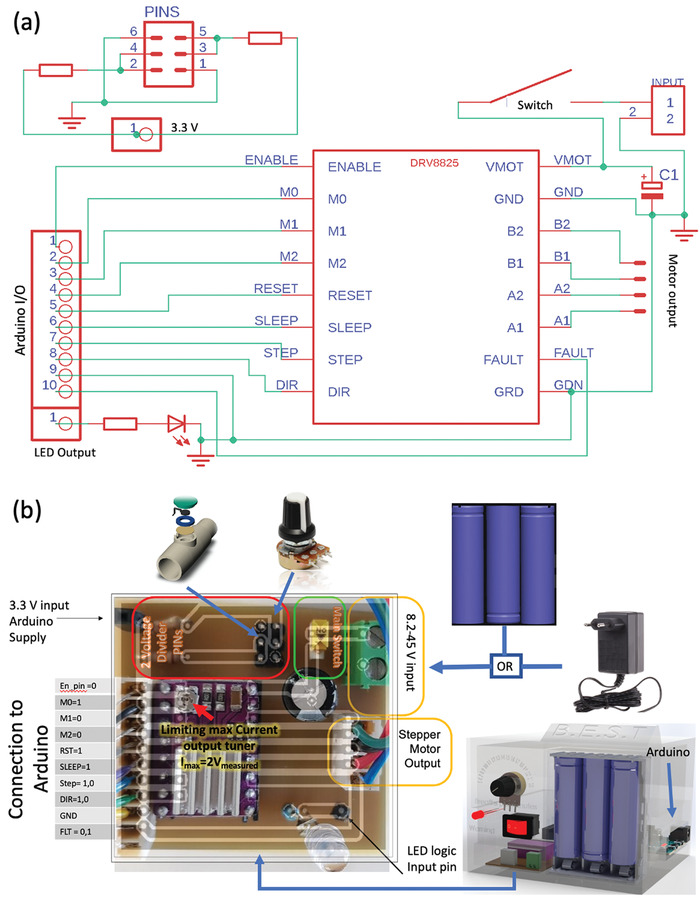
a) The schematic of custom‐made PCB for controlled operation of GlasVent and the b) developed PCB.

As mentioned earlier, GlasVent can be operated in three ways: The first scheme, i.e., manual operation, does not require any electronic component and accordingly the cost of the system is reduced to minimum. The second operation scheme (i.e., mains or battery operated) is the scheme with the most features. The heart of the system is the stepper motor driver printed circuit board (PCB) module (DRV8825 Stepper Motor Driver Carrier, High Current, Pololu electronics). A simple PCB was designed for driving the motor, as shown in the schematics in **Figure** **8**. All inputs and outputs of the DRV8825 are connected to the brain of the GlasVent system, i.e., Arduino Due. The system has a main rocker switch to switch on or off the system. The system also has a potentiometer to control the breaths per minute (BPM) to be supplied to the patient via BVM. A voltage divider is connected with the potentiometer and the output of the divider is connected to an analog pin of the microcontroller. The pressure is monitored by a commercial pressure sensor (MPXV4006, Freescale Semiconductor) located near the output of the BVM. Alternative sensors designed for similar specifications can also be used for this purpose.^[^ ^17^, ^18^, ^19^ ^]^ The pressure monitoring is critical for safe operation of the system. Pressure sensor is supplied by the 5 V output pin of the Arduino. The sensor outputs 0–5 V corresponds to pressure range of 0–6 Kpa. The analog pins of the Arduino Due have a range between 0 and 3.3 V, therefore a voltage divider was implemented to remap the output range of the sensor to acceptable voltage for the microcontroller. This is only necessary with microcontrollers that operate at 3.3 V input threshold (an alternative microcontroller, such as Arduino Uno does not require this configuration). An LED is placed to warn the user about a potential problem with the system. The Arduino is connected with a Personal Computer (PC) or tablet where the pressure data are displayed to allow the user to have a complete view of the system. Alternately, it is possible to provide a dedicated port for display on smartphone.

For better portability and safe backup operation, the system can be powered with three Li‐Ion battery cells. The stepper motor driver can be supplied from 8.2 up to 45 V. The Arduino microcontroller can be supplied also from a 7 to 12 V supply. The battery pack can provide 10.2 V near depletion and 12.6 V fully charged, the 0.6 V above the 12 V recommended power supply to the microcontroller can be drop by a diode. This configuration makes the system portable and extremely useful in areas outside the grid. The best use of the portable scheme is in vehicles (e.g., ambulance) or remote areas where mechanical ventilators are not available. The battery pack theoretically can supply the system for a little less than 4 h and can be further enhanced with recent discoveries in power storing and harvesting devices.^[^ ^20^, ^21^, ^22^ ^]^ That is sufficiently long for any ambulance converted vehicle to transfer a patient from his/her residence to a nearby hospital.

### 4.3 Software and Modes of Operation

The system has three modes—the simplest is the continuous mandatory ventilation mode which is commonly used for paralyzed or sedated patients. The system can monitor regulated pressure at constant intervals set be the clinician. This mode does not use any feedback from the patient. The second mode is the assist control in which the patient could trigger the ventilator to receive the next breath. In the current version of the pressure sensor in GlasVent, the capability to sense below the atmospheric pressure relies on the PEEP valve. In case the patient does not trigger the next breath, the system is programmed to give the next breath according to the preset BPM set by the clinician from the BPM knob. If pressure detected by the sensor overcomes the safety limit, the system disables the motor immediately and the warning LED is turned on. The third mode of operation for GlasVent is the pressure support ventilation. In this mode, the system delivers a constant amount of pressure at the inhalation period. The system counts the steps taken by the stepper motor and can convert the steps into volume delivered. However, in this mode there are chances of stepping loss of the stepper and this may lead to zero compensation. However, this could be easily overcome in upgraded versions of GlasVent by using an encoder to track the stepping loss and correct the system accordingly. A practical problem could be related to underestimating the need of TV from the user as the TV max is set physically.

### 4.4 Materials, Fabrication, and Assembly

The GlaVent is designed keeping in view the potential disruption of materials in the supply chain as several countries in lockdown. In that regard, the design features simple shapes with holes for bolts and nuts without the requirement of gears or belts, which complicate the assembly. The majority of the structure is made of acrylic sheets (3 mm thick and 6 mm thick), specifically a large sheet of acrylic was used as a base for the rest of the parts, the brackets that hold the BVM and the slider guides of the manual adjustment mechanism. The transparent casing made of acrylic allows a person to see the moving parts of the ventilator, which is psychology important as it gives confidence about working of the system. The acrylic was cut by a laser cutter, however other materials and tools, such as blade cutter or machining could be used too. The moving parts were 3D printed with polylactic acid filament with printing parameters similar to those explained elsewhere.^[^ ^21^ ^]^ The system also utilizes two steel rods (6 mm dia) to hold moving parts. Most of the 3D printing materials could be fabricated via different methods depending on the available tools. The electronic components used are simple and available in abundant supply and housed in a 3D printed developed in similar ways as explained elsewhere.^[^ ^21^, ^23^ ^]^ Further, these components are through‐hole, which removes the need for a dedicated PCB as they can be assembled with just a breadboard or a Veroboard. The assembly of GlasVent system is easy and requires basic tools, such as screwdrivers which are easily available and thus facilitate the DIY concept. The complete assembly of GlasVent system requires 1–2 h. The assembled system has been tested for long operation on a medical anatomic mannequin, which clearly showed the expansion for artificial lungs (see Videos S1 and S2, Supporting Information).^[^ ^24^ ^]^

## 5 Benefits, Opportunities, and Challenges for Emergency Ventilators

The first use of ventilators in modern medicine was reported to have been started by Bjorn Ibsen in Copenhagen in 1953, whose use of mechanical ventilation helped to save scores of lives of patients with polio, reducing mortality rate from 87% to 25%. In pre‐COVID‐19 days, ventilators were commonly used in patients with issues, such as chronic lung disease, severe asthma, chronic bronchitis, or pulmonary fibrosis, etc. This means that there is a long‐term, growing need for mechanical ventilation due to myriad aetiologies. This is further supported by the ageing demography—more than half of intensive care unit (ICU) patients in the United States are over the age of 65, a demographic group which is expected to grow from 46 million in 2014 to 74 million by 2030. Similar trends in Europe and Asia reflect this worldwide problem. To meet the growing demand for acute clinical care, ICUs will need to increase their capacity as well as their capabilities. Furthermore, simplified versions of ventilators may have a domiciliary role for intermittent support in chronic respiratory conditions, with the doctor's guidance via telemedicine, such as asthma or chronic degenerative/fibrosing lung conditions. They may be adaptable to aid delivery of inhaled medications, or to reopen blocked terminal airways. In such case, the ventilators could also be monitored remotely for oxygen concentration, frequency of use, etc. Thus, increased ventilator availability may help the clinical care of a wider variety of patients than COVID‐19 cases, including patients with injury, pulmonary noncommunicable diseases, and communicable ones, such as the human immunodeficiency virus and malaria.^[^ ^25^ ^]^ The need for mechanical ventilatory support is of short duration in a majority of patients, for example during postsurgical recovery, infectious diseases, trauma resuscitation, and in prehospital care. In many of these settings there is a potential role for a robust, simple, ventilatory assist device, particularly to augment capacity in LMICs (low‐ and middle‐income countries) or early phase epidemics of respiratory diseases. These devices could also have additional capacity enhancing roles, during epidemics, such as COVID‐19, to release advanced ventilators from use in low‐specificity indications.

Although the automated versions of manual resuscitator here cannot replace the clinical care provided by conventional mechanical ventilators, an AMBU bag as the backbone of a rapidly deployable emergency DIY ventilator has some merits, not least that most healthcare facilities have significant stock. Further, the AMBU bags have already been tested and certified for medical use, making them suitable for sterilization, while they are also compatible with a range of other equipment available in hospitals (masks, valves, intubation equipment, filters, oxygen supply). Some BVMs also come with incorporated safety features (pop‐off valves, PEEP valves) thus simplifying the rest of the design. However, drawbacks exist for COVID‐19 use, including the risk of aerosolizing virus unless exhaled air filtration is added; most of the emergency ventilators designed so far (Section 2) suffer from this issue.

In resource‐poor settings, refugee settings, and some LMICs the low availability of mechanical ventilators may render these novel devices the patients’ only option. High purchase and servicing costs (US$ 20 000–100 000), and inadequate technical support systems, are a bottleneck for provision of medical ventilators in resource‐poor settings and LMICs.^[^ ^26^ ^]^ The much lower cost of basic emergency ventilators may render them applicable, even if their capability is not as extensive as their expensive counterparts, as long as ethical barriers are met regarding safety and regulatory approval.

Other barriers for ventilation in some part of world apply to both traditional mechanical ventilators and novel emergency devices, such as religious or cultural beliefs coupled with misunderstanding about treatment effectiveness. Use of any ventilator requires appropriately trained, medical or nursing staff (typically intensive care physicians, anaesthesiologists, intensive care nurses, and respiratory therapists), which are not always available. In many settings the ventilator must be synchronized with the patient's natural inhalation and exhalation efforts and be dynamically adaptable to provide the pattern of mechanical support that is optimal for the individual patient, disease, and phase of pathology. Mismatches between the patient's demand and the machine's delivery can result in (potentially fatal) iatrogenic barotrauma or cause a patient to “fight the ventilator” (e.g., if a patient naturally needs more time to exhale but the ventilator prematurely transitions to lung inflation). Artificial intelligence could address this issue, as it has in other areas and applications.^[^ ^27^ ^]^

There are still many outstanding barriers to the provision of safe mechanical ventilation, not least safely maintaining an airway seal, and dynamically defining, monitoring, and adjusting the optimal ventilatory parameters for any given patient over time. Trained medical staff are needed, along with secure and electricity oxygen supplies. Although the GlasVent overcomes some of these issues in the short terms, as it permits manual/battery powered ventilation, using commercial tablets/phones for its software resource, and monitoring/control displays, it is clear that considerable development work is needed to meet regulatory requirements, and to ethically deliver even basic ventilatory capacity within healthcare settings.

## 6 Conclusion

The need for ventilators has increased rapidly due to recent worldwide spread of coronavirus. As a result, many established manufactures of ventilators are reallocating resources to increase production of the essential equipment while also making some of their designs freely available. We are also witnessing a wave of initiatives focusing on producing low cost emergency ventilators suitable for fast deployment to meet short‐term needs gaps. Some of these have been discussed and compared here along with detailed description of GlasVent. None will match or replace existing medical‐grade ventilators, but they do merit attention and development in the current situation and have potential to impact non‐COVID‐19 needs requirements around the globe. To realize these opportunities further testing, development, and regulatory approval will be needed, along with systems to enable the definition of optimal ventilator protocols that can be delivered and refined by appropriately trained medical staff.

## Conflict of Interest

The authors declare no conflict of interest.

## Supporting information

Supporting Information

Supplemental Video 1

Supplemental Video 2
