# Supplementary material for: GlasVent—The Rapidly Deployable Emergency Ventilator
Source: Glob Chall. 2020 Sep 6;4(12):2000046. doi: 10.1002/gch2.202000046 (PMC7713554; doi:10.1002/gch2.202000046)
Supplement: Supplementary file 1 — Supporting Information [file GCH2-4-2000046-s001.pdf]

© 2020 Wiley-VCH GmbH

Global Challenges
Open Access

Supporting Information

for *Global Challenges*, DOI: 10.1002/gch2.202000046

GlasVent—The Rapidly Deployable Emergency Ventilator

*Adamos Christou, Markellos Ntagios, Andrew Hart, and Ravinder Dahiya**
